# Contralateral transvenous left ventricular lead placement of an implantable device with pre‐sternal tunneling in an ipsilateral chronically obstructed subclavian vein

**DOI:** 10.1002/ccr3.9321

**Published:** 2024-08-10

**Authors:** Ibrahim Antoun, Sotirios Dardas, Islam Mubashwirul, Abu‐Tariq Taher, Nauman Ahmed

**Affiliations:** ^1^ Department of Cardiology Royal Derby Hospital Derby UK; ^2^ Department of Cardiovascular Sciences University of Leicester Leicester UK

**Keywords:** cardiac resynchronization therapy, device upgrade, implantable cardiac devices, lead tunneling, venous occlusion

## Abstract

Multiple methods are used to tackle ipsilateral obstructed venous access in patients undergoing a device upgrade by implanting a new left ventricular lead. One feasible solution to tackle this is implantation of the upgrade lead contralaterally with pre‐sternal tunnelization to the opposite side under conscious sedation.

## 1 INTRODUCTION

Cardiac resynchronization therapy (CRT) by biventricular pacing is now established as an adjunct to optimal medical treatment in patients with symptomatic heart failure with reduced ejection fraction and intraventricular conduction delay. This can be achieved by implanting a whole system at once or as an upgrade on a previous pacemaker with a new left ventricular (LV) lead implantation. Though uncommon, pacemakers cause problems long after the original implantation. Examples of late complications include pacemaker‐induced cardiomyopathy, lead failure, and venous occlusion, which can be partial or complete. Complete venous occlusion (CVO) has been reported as high as 9% in the literature.^1^ Multiple methods were suggested to tackle this, which will be explained later. We describe the case of a patient with CVO related to previous dual‐chamber pacemaker implantation who required a CRT upgrade. A contralateral transvenous LV lead implantation and pre‐sternal tunneling were pursued under conscious sedation with satisfactory subjective and objective outcomes.

## 2 CASE HISTORY

Our case is of a 73‐year‐old man with previous implantation of a dual‐chamber pacemaker 8 years before the procedure of interest for symptomatic sick sinus syndrome. His past medical history includes stable angina, for which he takes bisoprolol 2.5 mg once a day (OD) and glyceryl trinitrate spray as needed. He also takes atorvastatin 40 mg OD. He is a previous smoker. He is not diabetic, and his lipid profile was normal 2 months before the procedure of interest. He denied any cardiac chest pain within the past 2 years. He had no previous history of venous thromboembolic events or peripheral artery disease. A transthoracic echocardiogram (TTE) at the time demonstrated preserved LV systolic function. Pacing checks were satisfactory throughout the follow‐up. Pacing checks during the next 8 years revealed 1%–3% atrial and 22%–27% ventricular pacing rates. He presented to our hospital with a clinical picture of decompensated heart failure.

## 3 METHODS

**FIGURE 1:**
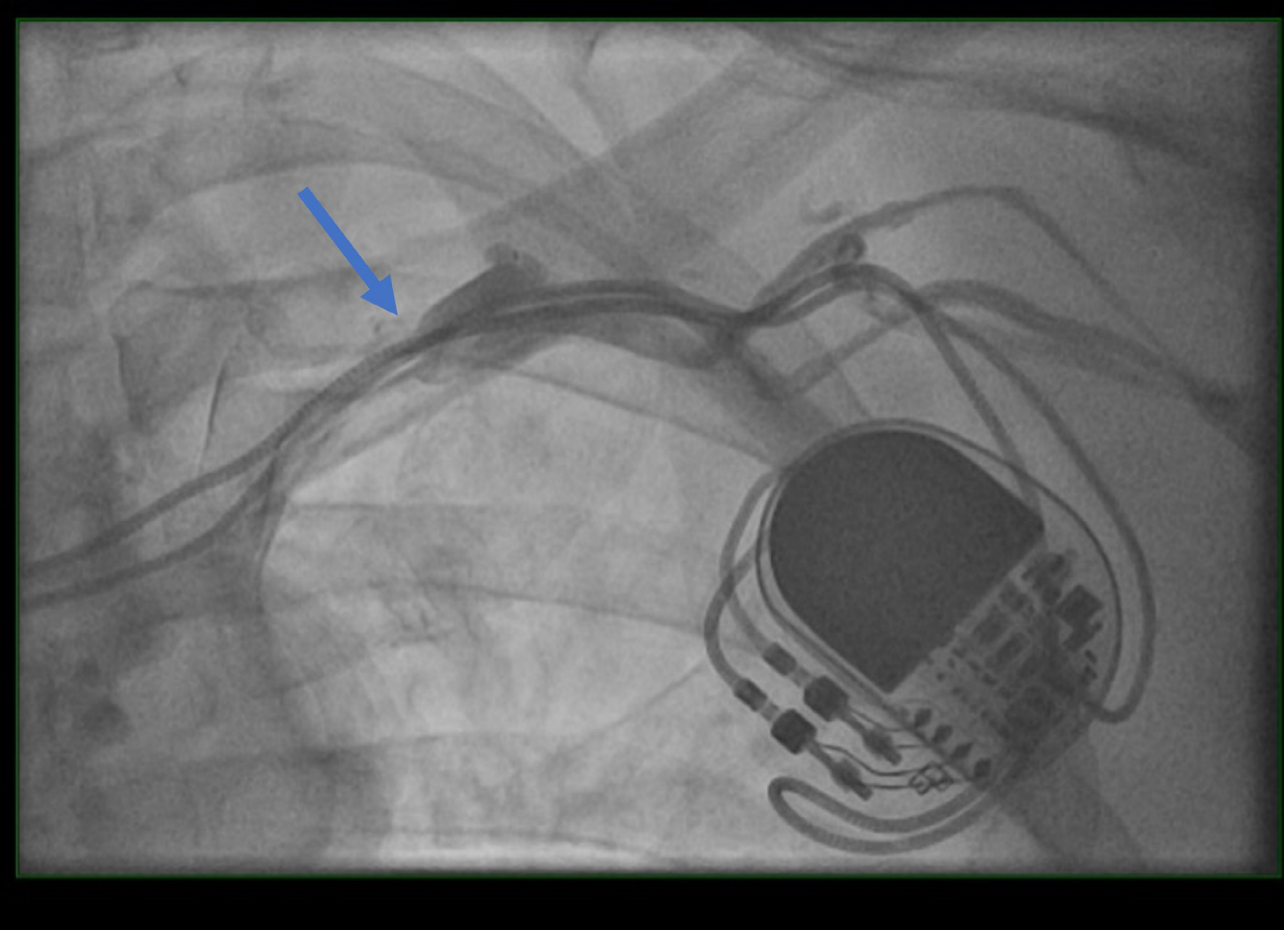
Left‐sided upper limb venogram conducted during the initial device upgrade procedure showing complete venous occlusion marked by the blue arrow.

A repeat TTE demonstrated deterioration of the previously preserved LV systolic function, with an estimated ejection fraction of 36%–40%. A 12‐lead electrocardiogram showed a paced rhythm. A consensus from our hospital's devices specialists proposed that the cause of the LV function drop is due to pacemaker induced cardiomyopathy. The proposed plan was to upgrade his device to CRT to improve dyssynchrony and his symptoms which happened 2 weeks after his initial presentation. During the upgrade procedure, an effort to advance a guide wire in the subclavian vein was unsuccessful due to CVO, as demonstrated with a left‐sided venogram (Figure 1). A right upper limb venogram in the same setting showed a patent right‐sided venous system. The procedure was then terminated, and the patient returned after 2 weeks for an alternative approach incorporating a tunneling technique. This technique was pursued because of the following reasons:

1. Minimally invasive technique compared to other surgical techniques.

2. Avoidance of complete lead extraction and contralateral implantation, which can lead to life‐threatening complications.

3. Maintaining a single pocket instead of creating a new one and increasing infection risk.

4. An experienced cardiac device doctor can perform a simpler technique in a shorter procedure time, especially under local anesthesia.

5. Patient preference after discussing the risks and benefits of this approach compared to new system implantation and other surgical approaches explained later.

**FIGURE 2:**
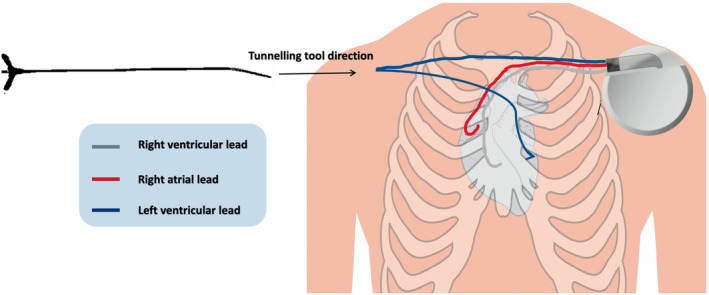
Demonstration of the anatomy of the pre‐sternal lead tunneling approach. The tool used to perform the tunneling is marked by the blue arrow, and a malleable stainless‐steel tunneling tool (Medtronic 6996T, SPC: FQE1439) fed through the OptiSeal Valved PTFE Peelable Introducer (model 1000093‐011).

The procedure was done under conscious sedation. The right axillary vein was accessed, and a 9Fr safe sheath introducer kit (Medtronic Ltd‐Cordis CATH F6 ST+ AL II 100 cm) was deployed. A 92‐cm Abbot Quartet LV Lead was inserted in the anterolateral coronary sinus branch via a 190‐cm SION wire (Vascular Perspectives Ltd) with satisfactory pacing parameters. The LV lead was tunneled contralaterally using a malleable stainless‐steel tunneling tool (Medtronic 6996T), fed through an OptiSeal Valved PTFE Peelable Introducer (Figure 2). Together with the two old leads, the LV lead was connected to a Percepta Quad CRT‐P MRI SureScan device (Medtronic Ltd) on the left side. The device was covered with a TYRX Absorbable antibacterial envelope (Medtronic Ltd) and placed in the pocket. Both wounds were closed with Coated VICRYL™ Polyglactin 910 Sutures. Hemostasis was achieved at the end of the procedure without immediate complications.

## 4 RESULTS

**FIGURE 3:**
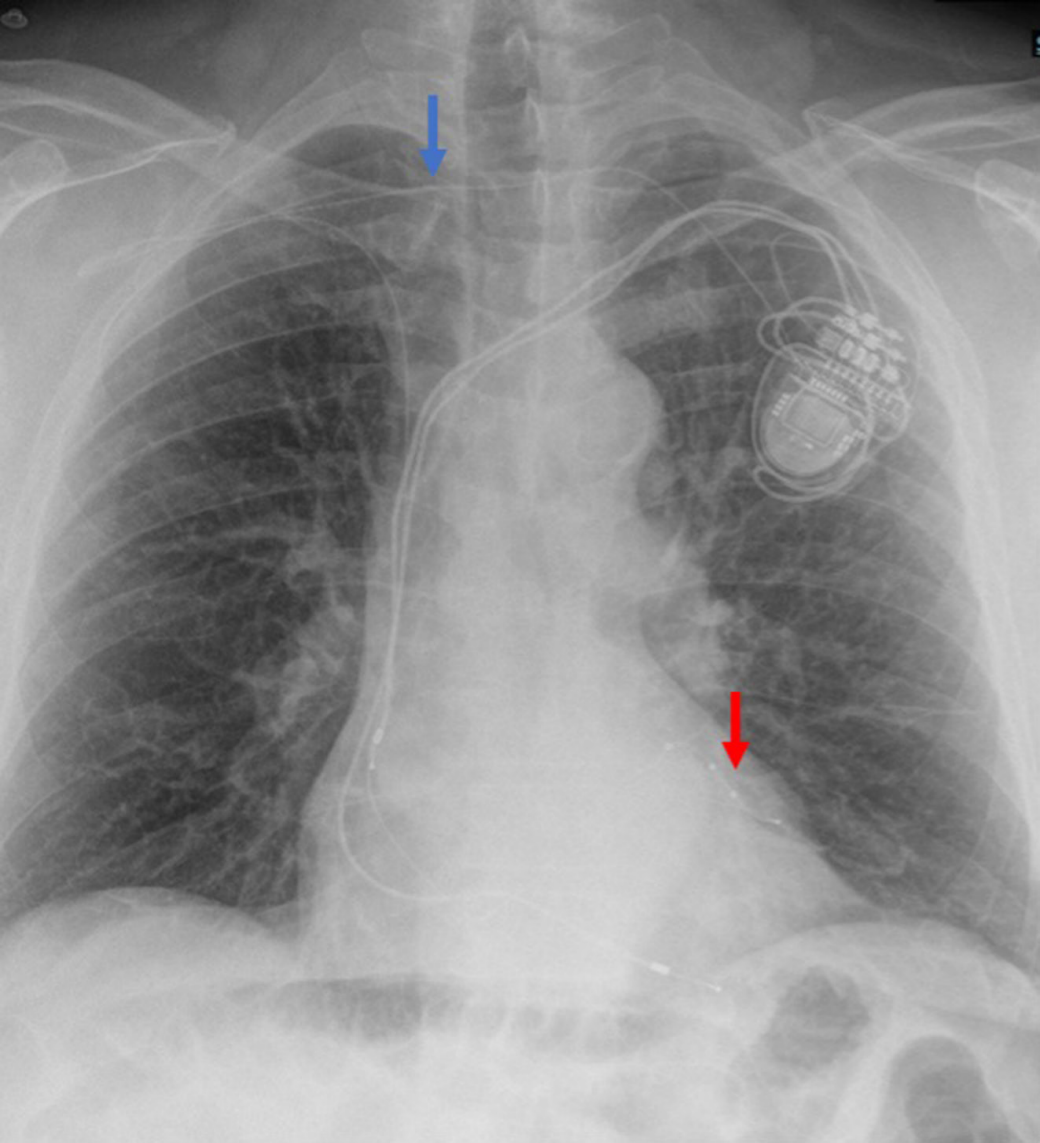
Posterior–anterior chest X‐ray after the device upgrade procedure demonstrates the tunneled left ventricular lead tunneling (blue arrow). The lead was implanted in the anterolateral coronary sinus branch (red arrow).

Post‐procedural pacing check showed normal LV impedance and threshold of 1.25 V, and chest X‐ray demonstrated satisfactory lead positioning without evidence of pneumothorax (Figure 3). There were no immediate complications. The patient was discharged home the same day. Subsequent follow‐ups with the patient in the pacing clinic showed satisfactory pacemaker function with a 98% biventricular pacing rate. A follow‐up echocardiogram 4 months afterward demonstrated an improved LV function to 50% by visual assessment with subjective symptomatic improvement.

## 5 DISCUSSION

Technological advancements and the growth of the aging population have led to an increase in the implantation rates of complex cardiac devices in recent years. At the same time, the rate of re‐interventions at the sites of original device implantation to either replace a failing lead or upgrade to CRT is rising, with up to 25% of patients requiring a repeat procedure.^1^

CVO can be encountered in up to 9% during these procedures and can be challenging to manage.^1^ Different methods have been adopted to overcome this relatively common problem. Ovadia et al. described an ipsilateral deep supraclavicular approach whereby access was gained to either the brachiocephalic or deep subclavian vein, followed by tunneling of the electrodes (pre‐ or retroclavicularly) to a pre‐ or retro pectoral pocket.^2^ A similar approach was reported by Antonelli et al., where access was gained medially to the occlusion via a puncture 1 cm lateral to the lateral head of the sternocleidomastoid muscle and 1 cm cranial to the clavicle. This approach mandates tunneling of the leads over the clavicle.^3^ Although both techniques were reported to be safe, they confer the theoretically increased risk of pneumothorax. Puncture of the contralateral innominate vein with subcutaneous tunneling of the leads has also been described as an alternative bail‐out strategy.^4^ Laser sheath extraction of non‐functional leads and recanalization of the occluded vein securing a route for new lead implantation is another potential approach, as is epicardial lead implantation via minimally invasive surgical procedures.^5^, ^6^ Although feasible, these approaches are more invasive and associated with a higher risk of complications. Similar is the case for the methods described by Lüthje et al. and Fox et al., where more aggressive techniques were used to tunnel the contralaterally implanted leads.^7^, ^8^ Sadarmin et al. described two cases in which they used a similar method to ours; however, in both cases, the procedure was done under general anesthesia.^9^

Although it is a relatively newer technique, we propose the following inclusion criteria for this cohort:

1. Documented ipsilateral venous system occlusion and a patent contralateral venous system.

2. Patients with a guidelines indication for device upgrade^10^ who have patent contralateral venous system.

3. Stable patients cardiovascularly without evidence of active infections.

4. Suitable anatomy for contralateral tunneling, including sufficient subcutaneous tissue and the absence of anatomical abnormalities.

To our knowledge, this is the first reported case where pre‐sternal tunneling of a contralaterally implanted transvenous LV lead was utilized under conscious sedation to overcome the problem of ipsilateral CVO.

## 6 CONCLUSION

LV lead pre‐sternal tunneling is relatively straightforward and uses a widely accessible kit. Although more data are required before this approach becomes widely applicable, our method seems feasible, safe, and relatively simple when dealing with CVO in patients who need a device upgrade.

## AUTHOR CONTRIBUTIONS

**Ibrahim Antoun:** Conceptualization; data curation; writing – original draft. **Sotirios Dardas:** Writing – review and editing. **Islam Mubashwirul:** Writing – review and editing. **Abu‐Tariq Taher:** Writing – review and editing. **Nauman Ahmed:** Writing – review and editing.

## FUNDING INFORMATION

This study has not been funded.

## CONFLICT OF INTEREST STATEMENT

There is no conflict of interest to declare.

## CONSENT

Written informed consent was obtained from the patient to publish this report in accordance with the journal's patient consent policy.

## Data Availability

Data relating to this study are available upon reasonable request from the corresponding author.
